# Immunological Outcomes of Allergen-Specific Immunotherapy in Food Allergy

**DOI:** 10.3389/fimmu.2020.568598

**Published:** 2020-11-03

**Authors:** Ann-Marie Malby Schoos, Dominique Bullens, Bo Lund Chawes, Joana Costa, Liselot De Vlieger, Audrey DunnGalvin, Michelle M. Epstein, Johan Garssen, Christiane Hilger, Karen Knipping, Annette Kuehn, Dragan Mijakoski, Daniel Munblit, Nikita A. Nekliudov, Cevdet Ozdemir, Karine Patient, Diego Peroni, Sasho Stoleski, Eva Stylianou, Mirjana Tukalj, Kitty Verhoeckx, Mihaela Zidarn, Willem van de Veen

**Affiliations:** ^1^ COPSAC, Copenhagen Prospective Studies on Asthma in Childhood, Herlev and Gentofte Hospital, University of Copenhagen, Copenhagen, Denmark; ^2^ Department of Pediatrics, Slagelse Sygehus, Slagelse, Denmark; ^3^ Allergy and Immunology Research Group, Department of Microbiology, Immunology and Transplantation, KU Leuven, Leuven, Belgium; ^4^ Clinical Division of Pediatrics, UZ Leuven, Leuven, Belgium; ^5^ REQUIMTE-LAQV, Faculdade de Farmácia, Universidade do Porto, Porto, Portugal; ^6^ School of Applied Psychology, University College Cork, Cork, Ireland; ^7^ Department of Paediatrics and Paediatric Infectious Diseases, Institute of Child’s Health, Sechenov First Moscow State Medical University (Sechenov University), Moscow, Russia; ^8^ Experimental Allergy Laboratory, Department of Dermatology, Medical University of Vienna, Vienna, Austria; ^9^ Division of Pharmacology, Utrecht Institute for Pharmaceutical Sciences, Faculty of Science, Utrecht University, Utrecht, Netherlands; ^10^ Centre of Excellence Immunology, Danone Nutricia research, Utrecht, Netherlands; ^11^ Department of Infection and Immunity, Luxembourg Institute of Health, Esch-sur-Alzette, Luxembourg; ^12^ Institute of Occupational Health of RNM, Skopje, North Macedonia; ^13^ Faculty of Medicine, Ss. Cyril and Methodius, University in Skopje, Skopje, North Macedonia; ^14^ Inflammation, Repair and Development Section, NHLI, Imperial College London, London, United Kingdom; ^15^ Institute of Child Health, Department of Pediatric Basic Sciences, Istanbul University, Istanbul, Turkey; ^16^ Division of Pediatric Allergy and Immunology, Department of Pediatrics, Istanbul Faculty of Medicine, Istanbul University, Istanbul, Turkey; ^17^ SPI—Food Allergy Unit, Département Médicaments et Technologies pour la Santé (DMTS), Université Paris Saclay, CEA, INRAE, Gif-sur-Yvette, France; ^18^ Section of Pediatrics, Department of Clinical and Experimental Medicine, University of Pisa, Pisa, Italy; ^19^ Regional Unit for Asthma, Allergy and Hypersensitivity, Department of Pulmonary Diseases, Oslo University Hospital, Oslo, Norway; ^20^ Children’s Hospital, Department of Allergology and Pulmonology, Zagreb, Croatia; ^21^ Faculty of Medicine, University of Osijek, Osijek, Croatia; ^22^ Catholic University of Croatia, Zagreb, Croatia; ^23^ Division of Internal Medicine and Dermatology, University Medical Center Utrecht, Utrecht, Netherlands; ^24^ University Clinic of Pulmonary and Allergic Diseases Golnik, Golnik, Slovenia, Faculty of Medicine, University of Ljubljana, Ljubljana, Slovenia; ^25^ Swiss Institute of Allergy and Asthma Research (SIAF), University of Zurich, Davos, Switzerland; ^26^ Christine Kühne-Center for Allergy Research and Education (CK-CARE), Davos, Switzerland

**Keywords:** food allergy, immunotherapy, tolerance, outcome measures, oral immunotherapy, immunology

## Abstract

IgE-mediated food allergies are caused by adverse immunologic responses to food proteins. Allergic reactions may present locally in different tissues such as skin, gastrointestinal and respiratory tract and may result is systemic life-threatening reactions. During the last decades, the prevalence of food allergies has significantly increased throughout the world, and considerable efforts have been made to develop curative therapies. Food allergen immunotherapy is a promising therapeutic approach for food allergies that is based on the administration of increasing doses of culprit food extracts, or purified, and sometime modified food allergens. Different routes of administration for food allergen immunotherapy including oral, sublingual, epicutaneous and subcutaneous regimens are being evaluated. Although a wealth of data from clinical food allergen immunotherapy trials has been obtained, a lack of consistency in assessed clinical and immunological outcome measures presents a major hurdle for evaluating these new treatments. Coordinated efforts are needed to establish standardized outcome measures to be applied in food allergy immunotherapy studies, allowing for better harmonization of data and setting the standards for the future research. Several immunological parameters have been measured in food allergen immunotherapy, including allergen-specific immunoglobulin levels, basophil activation, cytokines, and other soluble biomarkers, T cell and B cell responses and skin prick tests. In this review we discuss different immunological parameters and assess their applicability as potential outcome measures for food allergen immunotherapy that may be included in such a standardized set of outcome measures.

## Introduction

Food allergy (FA) affects a large number of children and adults throughout the world, with more than 170 foods being reported to cause food-induced allergic reactions. The development of effective treatments, besides avoidance of food allergens and the use of self-injectable epinephrine, is usually considered a top priority for patients and their families, advocacy groups, funding agencies, and research teams (1, 2).

Allergen-specific immunotherapy (AIT) is a treatment option for immunoglobulin E- (IgE) mediated allergic diseases, including FA. Food allergen immunotherapy (FA-AIT) involves administration of increasing doses of a specific food until achievement of maintenance dosage (1). So far, researchers and clinicians have focused on four major application routes of AIT, namely oral immunotherapy (OIT), sublingual immunotherapy (SLIT), subcutaneous immunotherapy (SCIT) and epicutaneous immunotherapy (EPIT) for the treatment of food allergies. In most cases, different routes and/or doses of AIT delivery results in similar immunologic changes (3, 4). During the last decade, OIT, SCIT, and SLIT have been widely evaluated in clinical trials for FA-AIT, while EPIT has been predominantly tested in a preclinical setting (1, 5) until very recently when well-powered clinical trials began to occur (6). Before, during and after FA-AIT, an oral food challenge (OFC), either in open or double-blind placebo-controlled (DBPCFC) format, can be used to assess the allergen reactivity threshold and functional tolerance (7). The cellular and molecular mechanisms that determine successful change in threshold and/or sustained unresponsiveness (SU) following long-term AIT in patients with FA are not fully understood, and FA-AIT is often accompanied by adverse reactions. Therefore, improvement of the treatment protocols, application routes, and relevant endpoints is warranted (1, 2).

Meta-analysis of different FA-AIT clinical trials is often challenging because of the lack of consensus on the outcome measures that are recorded. Our understanding of the cellular and molecular mechanisms that take place during FA-AIT would strongly benefit from standardized recording of a set of clinical and surrogate endpoints in all FA-AIT trials.

This comprehensive review provides an overview of the various immunological outcome measures for FA-AIT. The data that are discussed in this paper are primarily derived from the human clinical trials for FA-AIT (OIT, SLIT, SCIT, and EPIT), with the aim to summarize the most important immunological parameters that have been measured in FA-AIT clinical trials, and to assess their value as potential biomarkers for outcome prediction and disease monitoring in FA-AIT.

## Immunological Mechanisms of IgE-Mediated Food Allergy

Allergic diseases are the result of TH2 skewed immune responses to environmental antigens or allergens. In the case of IgE-mediated FA, these allergens are typically food proteins. The first step in the development of allergic disease is allergic sensitization, during which allergen-specific T and B cells become activated, clonally expanded and differentiated (8). Allergic sensitization can occur through different routes of exposure to food allergens. First reactions to common food allergens such as peanut and tree nuts often occur after the first ingestion, and previous research indicates that primary sensitization through non-oral routes is not unusual (9).

Most of our knowledge on the mechanisms of allergic sensitization and tolerance to food allergens comes from mouse models. Oral exposure typically induces tolerance but may also result in sensitization, especially when barrier-damaging adjuvants such as staphylococcal enterotoxin B are present (10). Allergen exposure through skin and respiratory routes can also lead to sensitization to food allergens (11–13). The relation between food allergies and an impaired skin barrier is also illustrated by high prevalence of food allergies among atopic dermatitis patients, particularly with severe forms (14, 15).

Food proteins can pass the intestinal epithelial barrier through transcytosis, paracellular diffusion or endocytosis by microfold (M)-cells (16, 17). In addition, intestinal epithelial cells can express major histocompatibility complex (MHC)-II molecules and thus may directly present allergen-derived peptides to CD4+ T cells in the gut. Food proteins can also be captured through transluminal processes by CX3CR1+ antigen presenting cells (APCs) that can sample intestinal antigens by extending transepithelial dendrites into the gut lumen. These CX3CR1+ APCs appear to be non-migratory, unable to activate naïve T cells and remain in the intestinal epithelium (18). These cells may be able to transfer antigens to migratory dendritic cells (DCs) that are present in the intestinal mucosa.

A healthy response to food antigens is characterized by immune tolerance, which is driven by DC-mediated antigen presentation in the gut. CD11c+CD103+ DCs are enriched in the Peyer’s patches of the intestine. Upon antigen uptake, these DCs can migrate to local lymph nodes where they exert classical DC functions and drive adaptive responses to food antigens (18). Dermal CD11b+ DCs as well as Langerhans cells play a central role in the induction of tolerance to allergens in the skin (19–21).

**Figure 1:**
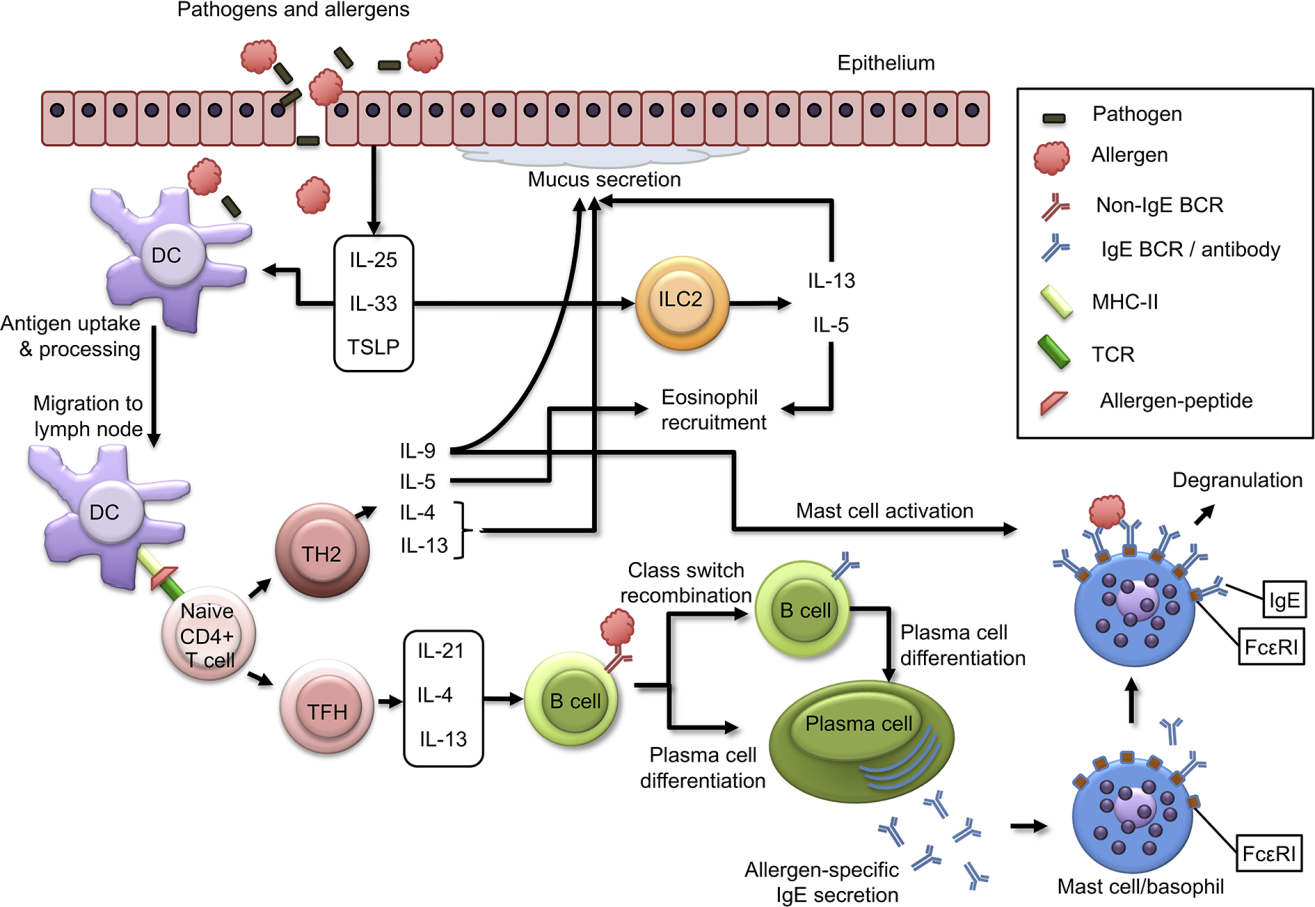
Immunological mechanisms of allergic sensitization. Epithelial cells produce alarmins such as IL-25, IL-33, and TSLP in response to external insults. These cytokines are central regulators of type 2 immunity, as they act on dendritic cells to induce TH2 responses and activate ILC2 cells leading to production of IL-13 and IL-5. Allergens and pathogens that pass skin or mucosal epithelial barriers are captured and processed by dendritic cells (DCs), which in turn migrate to draining lymph nodes, where they present allergen-derived peptides on MHC class II molecules to naïve T cells, which in turn (depending on co-stimulatory and cytokine signals) can differentiate to TH2 cells or TFH cells. TH2 cells produce type 2 cytokines such as IL-4, IL-13, IL-5, and IL-9, and function as effector cells that drive many aspects of allergic inflammatory responses. TFH cells produce IL-21, IL-4, and IL-13 and induce IgE class switch recombination in B cells, plasma cell differentiation and allergen-specific IgE production. Plasma cell produce allergen-specific IgE antibodies that are released into circulation and can bind to FcϵRI molecules on mast cells and basophils. Subsequent exposure to allergen can result in mast cell and basophil degranulation.

As a result of tissue injury and inflammation, epithelial cells produce TH2-inducing cytokines such as IL-25, IL-33, and thymic stromal lymphopoietin (TSLP). These cytokines act on a variety of cells involved in TH2 responses including DCs, mast cells, basophils, and innate lymphoid cells (22). IL-33 is critical for upregulation of OX40 ligand (OX40L) on DCs in a murine model of oral sensitization using cholera toxin, leading to TH2 differentiation of naïve T cells (23). When DCs that are conditioned by these epithelial-derived factors, capture allergen and migrate to lymph nodes, they interact with cognate T cells leading to clonal expansion and differentiation to TH2 cells, which are important effector cells that drive and perpetuate allergic responses. TH2 cells are polarized towards production of a distinct set of cytokines, including IL-4, IL-13, IL-5, and IL-9 (8). Of note, it still remains a matter of debate whether IL-9-producing CD4+ T cells are a distinct TH cell subset called TH9 cells, or rather a subpopulation of TH2 cells (24, 25). IL-4 and IL-13 are structurally and functionally related cytokines that play a central role in allergic inflammation through induction of IgE class switch recombination, smooth muscle cell contraction, goblet cell hyperplasia and mucus production (26). IL-5 plays a central role in allergic inflammation through eosinophil recruitment (27). While IL-5-mediated eosinophilic inflammation has been clearly demonstrated in certain asthma phenotypes, IL-5- and eosinophil-associated inflammation is less apparent in IgE-mediated food allergies (28). Interestingly, allergen-specific IL-5+ TH2 cells were only found in allergic eosinophilic gastroenteritis patients, whereas peanut allergy was associated with a dominant IL-5- TH2 response, indicating that heterogeneity within TH2 responses may favor IgE-mediated or eosinophil-dominant gastrointestinal inflammation (29). IL-9 contributes to allergic disease through, mucus secretion and chemokine release by epithelial cells, and mast cell proliferation (30, 31) (**Figure 1**).

While TH2 cells have long been considered to be critical for induction of IgE production by B cells, it has become increasingly apparent during the last decade, that IgE production by B cells requires interaction between B cells and follicular T helper (TFH) cells rather than TH2 cells (32). Via secretion of mediators such as IL-21, IL-4, and IL-13, TFH cells orchestrate B cells to differentiate to plasma cells producing allergen-specific IgE, which can bind to surface FcϵRIs on mast cells and basophils, where crosslinking upon re-exposure to the allergen will result in a type-I hypersensitivity reaction culminating in the clinical manifestations of food allergy (33) (**Figure 1**). Interestingly, a recent study demonstrated that the production of high affinity IgE anaphylactogenic antibodies is dependent on a subset of TFH cells, called TFH13 cells, which produce IL-13 in addition to IL-4 and IL-21 (34).

AIT has a wide range of effects on many components of the immune system. These include: I) an early decrease in mast cell and basophil activation (this can be observed within hours and can be maintained for months after the start of AIT (35, 36), II) generation of allergen-specific regulatory T cells (Tregs) and regulatory B cells (this can be observed within weeks to months), III) changes in allergen-specific antibodies often characterized by an temporary increase followed by a decrease in specific IgE and a gradual increase in specific IgG4 (37).

AIT in food allergy either delivered as OIT, SLIT, SCIT, or EPIT is done with the aim to desensitize the patient in order to raise the threshold for allergic reactions with the ultimate goal of achieving SU, which for e.g., peanut OIT has been demonstrated in some of the treated patients (38), however, long-term clinical efficacy still remains largely unknown (39).

## Food Allergen Immunotherapy in Clinical Trials

Over the past years, many patients have participated in randomized clinical trials (RCT) of FA-AIT of which OIT is the most widely used. Comparison of these clinical studies and interpretation of the results are difficult, primarily due to lack of standardization in protocols, dosing regimens and endpoint analysis (38, 40, 41).

Efficacy in RCT and effectiveness in real life studies has typically been determined by induction of a desensitized condition. Desensitization is defined as a state of temporary food allergen hypo-responsiveness or improvement in food challenge outcomes after therapy and an increased threshold for reactions compared with the pre-OIT threshold. Desensitization also refers to a certain degree of protection from anaphylaxis caused by unintentional ingestion of small amounts of a food allergen. Desensitization can be lost when OIT dosing is interrupted by nonadherence (irregular intake of doses) (42).

In assessing the clinical impact of food immunotherapy, it is important to distinguish desensitization from SU, remission, and oral tolerance. SU refers to a lack of a clinical reaction to a food allergen after an active therapy has been discontinued for a period of time, while remission refers to a temporary condition of non-responsiveness after active immunotherapy has been completed. Finally, oral tolerance is defined as a complete lack of clinical reactivity to a specific food allergen, as found in the natural development of tolerance (43). In practice, SU is often used as a substitute for permanent tolerance when reporting results of clinical trials. It is unclear what time period defines permanent tolerance to a specific food.

Comparison and interpretation of published clinical studies is not only adversely impacted due to terminological differences, but also due to the lack of criteria for evaluating clinical effects. The latter is complex and often lacking. In fact, efficacy has typically been measured in clinical trials using oral food challenges, therefore it is not yet known whether or not desensitization rates can protect patients from accidental exposure in real-life settings or can prevent severe or life-threatening allergic reactions or death (44).

Cow’s milk (CM), egg and peanut immunotherapy desensitize approximately 60 to 80% of patients studied (45–47). A majority of patients treated with food allergens through application of immunotherapy are at least partially desensitized, but this desensitization does not translate into decreased allergic reactions in real-life settings (48). It appears that the desensitization rates for other foods, e.g. wheat, sesame, hazelnut are not as high as those for CM, egg or peanut, but knowledge is lacking due to a paucity of clinical studies (49, 50).

SU has not been adequately studied to provide conclusive data. CM and peanut have been found to induce SU in 30%–70% of patients (43, 45). Interpretation of these data is difficult, as it is mainly based on a number of unspecific variables such as the patient’s age, delivery method, duration of active phase of immunotherapy, length of time off therapy, etc. OIT and EPIT, in their current form, are unlikely to induce a permanent or long-lasting measurable immunological and clinical response (47, 51).

A recent meta-analysis of clinical RCTs analyzed more than a thousand patients treated with peanut OIT followed up for a median of 12 months and estimated that 40% of the active group became desensitized compared with 3% of patients in the control group. However, significant risks of side effects or severe adverse events were observed in the treatment group (22%) versus the placebo group (7%) (47), including use of adrenaline or reactions defined as anaphylaxis. Furthermore, there was no evidence that OIT with peanut significantly improved quality of life in the participants compared with patients who continued to avoid peanuts.

The ﬁrst phase 3, placebo controlled, RCT of OIT was published in 2018. It included 551 patients aged 4–55 years, which responded at a maximum dose of 100 mg of peanut protein before starting (38). The primary endpoint was tolerating 600 mg of peanut protein in a DBPCFC. Results showed that 67% of the actively treated group tolerated 600 mg of peanut protein, versus 4% of the placebo group (p<0.0001). The incidence of serious adverse events was signiﬁcantly higher in the active group (4.3% vs. 0.8% of the placebo group).

Based on these results, the U.S. Food and Drug Administration on January 31, 2020 approved PALFORZIA™ [Peanut (*Arachishypogaea*) Allergen Powder-dnfp], a standardized OIT product for peanut allergy. The patient needs to carry an autoinjector of adrenaline at all times and initial dosing and up dosing phase has to be performed at a facility capable of treating severe allergic reactions, and patients have to go through a Risk Evaluation and Mitigation Strategy before starting therapy. PALFORZIA is recommended in combination with peanut avoidance and is contraindicated in individuals with uncontrolled asthma, eosinophilic esophagitis, and eosinophilic gastrointestinal disorders (38).

Sublingual immunotherapy (SLIT) may represent a safer therapeutic option for patients with food allergy, especially for those who want to protect themselves against more severe allergic reactions. Some studies showed that during SLIT, a statistically signiﬁcant desensitization rate in comparison with placebo was observed, however SLIT usually do not allow to reach the same change in threshold as OIT. The median threshold dose during SLIT increases approximately 20-fold, in comparison with more than 300-fold with OIT. Although not as effective, an advantage of SLIT over OIT is the higher safety proﬁle (52, 53).

Epicutaneous application of food allergens by patches that release small amounts of food protein *via* the skin is also a potentially safe delivery method of food allergen immunotherapy. EPIT requires application of the patch to intact skin to ensure a tolerogenic effect (6, 54, 55). The “Viaskin^®^ Peanut’s Efficacy and Safety” (VIPES) study and CoFAR 6 study were parallel phase 2 studies designed to investigate the efficacy and optimal dose of EPIT for peanut (54, 55). In the VIPES study, a statistical difference in responder rate was seen with the 250 µg but not with 50 or 100 µg patch. In the CoFAR 6 study, a statistically significant difference was found in both the 250 and 100 µg patch, but efficacy appeared to be stronger for the 250 µg patch. Local mild-to-moderate skin reactions were common, but only 0.9% in the VIPES study and 3.4% in the CoFAR 6 study withdrew due to local skin reactions.

Other approaches under investigation include OIT with multiple foods, OIT combined with biologics (omalizumab, dupilumab) or probiotics, and OIT with altered allergens, all with the aim of improving efficacy and reducing adverse events (56–58). So far, published studies have only been conducted in small patient samples.

Despite the risk of developing adverse events including severe allergic reactions and anaphylaxis, some patients may be motivated to attempt OIT rather than to continue using avoidance alone, because OIT has been found to mitigate the anxiety of severe allergic reactions in the event of accidental ingestion of specific food allergens (59). For such patients, it is important to provide accurate and factual information based upon the available data, not only on effectiveness, but also on the risk of adverse events. If food immunotherapy is undertaken, appropriate safety precautions, and ongoing communication with patients (and with parents/caregivers) are crucial.

## Functional Immunological Tests

### Skin Prick Test

Diagnosing FA can be challenging as symptoms can arise from different organ systems, vary in severity and should be often differentiated from other diseases. Therefore, an objective diagnostic measurement is important and useful in the diagnosis of FA. The skin prick test (SPT) is performed by applying droplets of standard allergen extracts and/or fresh foods, positive and negative controls on the volar forearm of the patient. A sterile lancet is used to prick through the droplet about 1 mm through the skin. The results can be directly assessed after only 15 min. A wheal ≥3 mm in diameter to at least one allergen is considered positive, in the context of a wheal ≥3 mm to histamine (positive control) and no wheal to the negative control. SPT is widely used in FA diagnostics and research, however, self-reported FA incidence is normally higher than SPT-diagnosed incidence (60).

Smaller SPT wheal diameter at baseline is considered a predictor of successful desensitization. A retrospective study of 100 patients (<18 years) who underwent at least 6 months of hazelnut OIT, revealed an association between successful desensitization and smaller hazelnut SPT wheal diameter at baseline (61). A similar pattern was also reported in a study involving 82 patients (2–18 years) who underwent CM OIT for 5 years (62). In contrast, a study of 120 patients (7–55 years) who underwent peanut OIT for a period of 2 years found no association between SPT wheal diameter at baseline and treatment success (63), which could be explained by the recruitment of patients with SPT wheal ≥ 5 mm and not the ≥ 3 mm conventionally interpreted as positive.

Emerging data suggest that SPT diameter seems to decrease during AIT. In a study involving 48 patients (21–38 years) with systemic allergic reactions to peach and/or peanut, from which 36 were treated with Pru p 3 SLIT for 12 months and 12 were non-treated (control group), 91% of the treated patients were successfully desensitized (64). The clinical efficacy of SLIT was evaluated by SPT and DBPCFC to peach or peanut at baseline and after 12 months of Pru p 3 SLIT. After 12 months of SLIT, treated patients presented significantly decreased peach or peanut SPT wheal area, as well as increased peach or peanut thresholds by DBPCFC. These results are also in agreement with other reports on fruit and peanut OIT available in the literature (42, 65, 66).

A peanut OIT involving 28 children (1–16 years), randomized to receive either peanut OIT (4000 mg) or placebo, found that peanut OIT was associated with increased peanut consumption compared to placebo after 12 months and decreased SPT size and TH2 cytokine levels as well as with increased peanut-specific IgG4 levels and regulatory T cells (Treg) (67). The findings were similar in a CM OIT trial involving 20 children with CM allergy (6–21 years) randomized to CM OIT (500 mg) vs. placebo OIT (68), and an egg OIT trial involving 55 children with egg allergy (5–11 years) randomized to egg OIT (2 g) vs. placebo OIT (69).

Maintenance therapy upon discontinuation of AIT may be used in order to prolong the achieved tolerance in patients with FA, but the maintenance doses vary greatly. Along with DBPCFC, SPT may be used to test the induced tolerance in patients undergoing OIT (53, 70–72).

Overall, OIT treatment success was associated with reduced SPT responses after ended treatment, and smaller SPT at initiation of treatment was predictive of better outcome of the OIT. In general, it is considered that the SPT provides an “*in vivo*” procedure for measuring the reactivity of sIgE -activated mast cells and basophils. A rapid decrease in the number of mast cells and basophils in skin, as determined by SPT, has been observed in immunotherapy for both aeroallergens (73) and food allergens (74).

### Mast Cell and Basophil Activation Test

Mast cells and basophils play an important role in allergies as major effector cells. Both cells have intracellular granules, in which biologically active mediators and cytokines are stored, which are released after activation (75). Mast cells are generally present in the skin, gastrointestinal and respiratory tracts. In the tissue, they reside as tissue resident cells and play a major role in the first defense against invading pathogens (76). Basophils form a small proportion (<1%) of leukocytes in the peripheral blood and enter the peripheral tissues only after activation during infection. Once activated, mast cells and basophils degranulate and release different mediators and cytokines, playing a role in defense against parasitic and bacterial infections (75, 77, 78).

For mast cell activation and/or degranulation, the most important cytokine receptors are the IL-4, IL-18, and IL-33 receptor (79), while the IL-18 and IL-33 receptor are also important for basophils (80). Both cell types express FcϵRI receptors, which play a major role in allergen recognition and subsequent induction of cell activation and/or degranulation (75). Binding of antigen by IgE bound on membrane-bound high affinity IgE receptor (FcϵRI), promotes cross-linking of at least two FcϵRI receptors, activating a signaling cascade (81, 82), which results in the release of different pro-inflammatory mediators (75). Some of these are present in the pre-formed granules and can be released within seconds or minutes after their activation, whereas others are *de novo* synthesized (81, 83). These mediators can be divided into different classes such as biogenic amines (e.g., histamine and basogranulin) proteases (e.g., tryptase, CPA3, and chymase), proteoglycans (e.g., heparin), lipid mediators (e.g., PAF, LTC4, and PGD2), cytokines (IL-4 and IL-13), chemokines (e.g., CCL2), peptides (e.g., endorphin), and other enzymes (e.g., β-hexosaminidase). Some of the basophilic and/or mast cell mediators have been associated with either (food) allergy or anaphylaxis. These mediators include histamine, tryptase, chymase, CPA3, PAF, PGD2, LTC4, basogranulin, heparin, and CCL2 (84–87). Besides secreted mediators also the expression of CD203c (identification marker) and CD63 (marker for anaphylactic degranulation), and phosphorylation of certain intracellular molecules (MAPK and STAT5) can be measured to assess basophil or mast cell activation (88).

The basophil activation test (BAT) is a functional assay in which patients’ whole blood or peripheral blood mononuclear cells (PBMCs), can be stimulated with the allergen. Basophils can also be enriched from the PBMC fraction by negative selection using magnetic beads. When the basophil is activated, different activation markers (CD63, CD203c, histamine) can be measured using flow-cytometry or fluorescent labelling (89). The whole blood assay is considered to be more physiologically relevant, because factors that are naturally present in the blood, such as blocking antibodies, may play a role in phenotype of allergic or non-allergic individuals (88). For the mast cell activation test (MAT), mast cells from a LAD2 mast cell line are incubated with patients’ sera and subsequently with the allergen. The degranulation of the mast cells is assessed by flow cytometry *via* measuring surface activation markers CD63 and CD107a, and/or mediator release, such as β-hexosaminidase and histamine (90). It has been shown that both BAT and MAT detect food allergy with a higher specificity than the SPT or sIgE measurements in serum, and could indicate severity of allergic reactions (88–91). However, both tests need further improvement, are still technically challenging (89, 90, 92) and not used in routine clinical practice. Basophils are more accessible than mast cells for *ex vivo* studies, which explains why most studies apply BAT.

Since no studies reported the use of MAT in FA-AIT we focused on BAT. There are numerous studies investigating BAT as a measurement of efficacy of AIT during peanut, CM, egg, peach, and apple OIT, SLIT, and EPIT (**Supplementary Table 1**), which generally show a reduction in the basophil activation. However, in only seven studies the BAT was one of the main outcome parameters (36, 93–99). Furthermore, correlation with the clinical outcome was only assessed in five studies (63, 94, 98–101). Here, we will focus on CD63, CD203c expression and histamine release as basophil activation markers, as well as on anti-IgE, fMLP, and IL-3 simulation on basophil function.

#### Basophil Marker CD63

Most studies used the CD63 activation marker for measuring basophil activation. A significant reduction in allergen-induced CD63 expression was observed in the majority of AIT studies including peanut (36, 53, 98, 101–104), CM (94, 100), and egg (95, 96, 105, 106) AIT.

In a large RDBPC phase 2 study (POISED study) 120 participants (7–55 years) were randomly assigned to peanut OIT (N=95) or the placebo group (N=25). After 12 weeks of OIT, the authors found a significant reduction in the %CD63^+^ basophils responding to anti-IgE alone or peanut (0–1000 ng), a number that continued to decrease until week 117 (99). Similarly, in most studies on egg AIT, a significant decrease of CD63 expression was seen after OIT with egg, and in general, most participants were tolerant after the OIT. A single center study with 28 egg allergic children (5–10 years) showed that OIT with egg white was effective. Twenty-seven out of 28 children became tolerant to egg and showed a significant decrease in CD63 expression to egg white, ovalbumin and ovomucoid (95). Another study with a combination of SLIT/OIT included 30 CM allergic children (6–12 years). The CD63 expression increased during SLIT build-up phase in those who did not become tolerant, and CD63 expression decreased after de second phase in those who became tolerant.

On the contrary, six studies showed no clear effect on basophil activation during AIT; three peanut (42, 54, 57), 2 CM (107, 108), and one apple (93). Importantly, five of these studies also showed no clinical effect as which correlates with little change in BAT. The one study showing effect, was a peanut OIT including 99 peanut allergic children (7–16 years) that showed a clear increase in peanut threshold after the OIT, but no significant within-patient differences after treatment for AUC or %CD63+ cells, although there was a reduction in %CD63+ cells at lower peanut concentrations after OIT (42).

In some cases basophil markers at baseline were excellent predictors for a higher tolerated dose in DPFC and sustained unresponsiveness after OIT (63, 94). The CM-omalizumab OIT study showed that CD63 expression of more than the threshold of 40% was likely to cause symptoms in the placebo group, but not in the omalizumab group (94). However, a CM OIT with 30 CM allergic children (100) showed that CD63, CD203c expression and histamine release at baseline could not predict outcome of AIT (tolerance). However, the study did reveal that CD63 and CD203c expression increased after SLIT build up phase in those who did not become tolerant. In subjects who became tolerant, CD63 expression was decreased, but the CD203c expression did not change.

Two peanut AIT studies investigated SU after a period of AIT followed by an avoidance period (98, 99). In both studies, a sustained reduction in %CD63+ cells was correlated to SU. In addition, one study showed, that SU after 13 weeks off OIT therapy was achieved primarily in two groups of subjects, those with lower basophil responses at study entrance and those who underwent a substantial reduction (80%–90%) of their peanut induced basophil activation after OIT (99). Furthermore, participants who failed the DBPCFC after initial OIT showed higher CD63 expressions at every time point (99).

Overall, it seems that the amount and duration of the maintenance dose as well as the type of IT (OIT vs. SLIT) could influence the basophil reactivity and subsequently the probability of sustained unresponsiveness.

#### Basophil Marker CD203c

Thirteen studies also assessed the basophil marker CD203c, including studies on peanut (36, 57, 98, 99, 101, 103, 104, 109), egg (105, 106), and CM (95, 100, 108). Only four studies showed a significant decrease in CD203c expression upon allergen stimulation during or after the AIT protocol (36, 99, 105, 106).

A multicenter randomized egg AIT study with 45 egg allergic children showed that the duration of the OIT is crucial for an effect on CD203c expression. No effect was found after 3 months of OIT, but after 12 months a reduction in CD203c expression and tolerance was encountered (105).

The remaining nine studies did not show a significant reduction of CD203c expression. In two CM-AIT studies no clinical effect was seen, which could explain the lack of responsiveness to the OIT of the CD203c expression (95, 108). The SLIT/OIT CM study, also described under CD63, showed that CD203c expression increased during SLIT build up phase in patients that did not became tolerant (comparable to CD63 expression), but in contrast to CD63 expression, CD203c expression did not change in those who became tolerant after OIT (100). This difference in CD63 and CD203c expression was also seen for a peanut OIT study with 120 peanut allergic patients (7–55 years) (99) and a SLIT/OIT peanut comparison study with 21 peanut allergic patients (6–21 years) (98). In both peanut studies CD63 expression decreased significantly after AIT while CD203c expression did not change. The four remaining studies only included the marker CD203c in their gating strategy while using CD63 as the primary marker for basophil activation (101, 104, 109, 110).

#### Histamine Release

Three studies measured histamine release; two with CM (94, 100) and one with peanut (98). The CM OIT study with omalizumab in 57 CM allergic patients (7–35 years) showed contradictory results between %CD63^+^ cells (decreased after omalizumab treatment) and histamine release (increased after omalizumab treatment). Histamine release was determined in the washed basophil-enriched fraction and %CD63^+^ in whole blood, which might be the reason for the differences between studies. The use of whole blood is preferred because the physiological *in vivo* conditions, such as the presence of blood components (e.g., IgG4, anti-IgE, complement factors), are better reflected. Moreover, the cells are less activated due to centrifugation and other handling steps (94). The SLIT/OIT CM study described under CD63 showed only a decrease in histamine release after SLIT/OIT treatment and not after SLIT/SLIT, while CD63 expression was reduced after both treatments. Also, in this study the histamine release was measured in a basophil enriched fraction (100). The peanut study compared a SLIT with OIT therapy in 21 peanut allergic patients (6–21 years). Histamine release was reduced in the OIT and SLIT groups compared with baseline, which was in line with the CD63 result. However, after the maintenance dose or off dose, the levels increased and where no longer significantly decreased in the OIT group. This study also used basophil enriched mononuclear cell fraction (98).

#### Anti-IgE, fMLP, and IL-3 Stimulation

Anti-IgE, N-Formyl-methionyl-leucyl-phenylalanine (fMLP), and IL-3 stimulation of isolated basophils is less frequently used to measure the responsiveness of the basophils at baseline and after immune therapy. Four studies used anti-IgE stimulation, including egg (95, 96), and peanut (36, 99), and only one study stimulated the basophils with IL-3 and fMLP to evaluate the effect of AIT (36). The OIT egg study with 28 egg-allergic patients (5–9 years) showed no significant differences in basophil activation levels after stimulation with anti-IgE before or after OIT, while 27 patients became tolerant to egg after the AIT (95). However, a smaller OIT study with only 10 egg allergic children (5–14 years) showed 30%–50% less reactive basophils after IgE stimulation and tolerance to egg was achieved in nine children. The randomized DBPC OIT peanut trial with 120 peanut allergic patients (7–55 years) showed that peanut OIT significantly reduced %CD63^+^ cells after stimulation with anti-IgE after 12 weeks. The second peanut OIT study with 28 peanut allergic children (2–8 years) showed that CD63 and CD203c expression on anti-IgE stimulated basophils was significantly decreased after OIT (36). A possible explanation could be that peanut OIT suppressed FcϵRI signaling. Upon stimulation with fMLP and IL-3, the basophil expression of CD203c, but not the expression of CD63 was suppressed after peanut IT. This suggests that IL-3 can directly induce the upregulation of basophil CD203c-expression, while FcϵRI triggering and fMLP receptor activation causes further upregulation of this marker (36).

#### Summary of Findings Related to Basophil Activation Tests

CD63 is expressed in several cell types, such as basophils, mast cells, macrophages and platelets, while CD203c is solely expressed in basophils. Moreover, basophils are primed with IL-3 to increase the sensitivity of CD63-based assays. In CD203c based assays, priming with IL-3 causes a non-specific increase in the test result. This might also explain the contradictory results on CD203c and CD63 expression. The difference in CD63 expression and histamine release might be related to the measurement of CD63 in a whole blood assay, while histamine release was assessed in the enriched basophil fraction. The use of whole blood mimics physiological *in vivo* conditions, such as the presence of blood components (e.g., IgG4, anti-IgE, complement factors) better. Overall, CD63 expression could be a suitable marker for basophil activation and for determining the efficacy of the AIT. However, the results were not always comparable, probably due to differences in the study design of the AIT, BAT, and blood collection. In summary, BAT may be a suitable objective measure of desensitization in AIT trials if the following factors are harmonized: type of allergen (extract or recombinant protein), concentration of allergen, use of IL-3 priming, type of biological sample (whole blood vs. enriched PBMCs), incubation time, staining protocol, and data analysis (%, AUC, SI, EC-50, CD-sens).

## Humoral Responses

### IgE

Since the discovery of the novel class of antibodies in 1966, IgE have been a hallmark of allergic disease (111). *In vitro* diagnosis relies on the specific binding of IgE (sIgE) to allergen molecules in order to determine the allergen source. However, the presence of IgE and the titers of sIgE do not normally correlate with the severity of the clinical reaction but rather represent reaction probability. Several factors related to IgE, such as antibody affinity, the ratio of sIgE to total IgE, the degree of antibody polyclonality, the number of epitopes recognized in one allergen as well as the number of allergens recognized in an allergen source, may all contribute to clinical reactivity in the patient.

sIgE has been reported to rise during the first few months of AIT without eventually leading to an increase of allergic symptoms (112). Upon prolonged immunotherapy, sIgE, similar to SPT wheal diameter, decreases over time. This observation seems to be independent of the administration route: oral, sublingual or epicutaneous. In a peanut OIT with 24 patients (1–16 years), sIgE to single components was shown to follow the same increase and decrease during the trial as sIgE to whole peanut extract (43). In addition, patients with a successful OIT (i.e. obtaining SU) had lower sIgE levels at baseline and at the end of the study, as well as a lower sIgE/total IgE ratio (43). The ratio of sIgE to total IgE has been analyzed in a large study of 161 children (11 months–18 years) in order to predict OFC outcome. Participants who failed OFC had a higher ratio of sIgE to total IgE than those who passed their challenge (113). Markedly, this ratio was significantly higher for food challenges related to more persistent allergies, such as peanut, tree nuts, shellfish, and seeds.

Total IgE has been studied in 3 CM OIT trials. Two studies reported no changes in total IgE during AIT (70, 114), and one found an increase at the end of therapy (68). One study of 23 patients (3–14 years) undergoing peanut OIT (115) and another DBPC study of 74 patients (4–25 years) undertaking peanut EPIT showed no change in total IgE during therapy (54).Two other studies (peanut SLIT and egg OIT) showed no difference in total IgE in responders compared to non-responders (53, 116).

More recently, a study of OIT for CM allergy in 24 children (13–22 years) also looked at potential biomarkers that could predict long-term outcome of OIT. They showed that total IgE measured at baseline and after 6 months of OIT proved to be not useful to determine responsiveness to this intervention (117).

The role of specific IgE-binding epitopes has not yet been thoroughly evaluated in the context of AIT. In a longitudinal study comparing 35 children (3–68 months) with persistent CM allergy to children that had naturally outgrown their allergy, it was shown that children with persistent allergy had greater intensity and broader diversity of IgE and IgG4 binding epitopes than children with transient CM allergy. In addition, children with transient CM allergy also had epitopes overlapping between IgE and IgG4 (118).

Ara h1, Ara h 2 and Ara h 3 peptide epitopes have been analyzed during a peanut OIT study including 22 patients (1–16 years). IgE peptide repertoire was broad at baseline and tended to diminish during therapy. Although sIgE levels decreased, some patients developed novel IgE specificities (119). Recently, a peanut OIT trail in toddlers revealed that IgE-binding to specific Ara h 2 epitopes, in addition to testing peanut extract, is useful for stratifying patients, both in regard to food challenge sensitivity prior to OIT and to prediction of OIT outcome (120). In another study of CM OIT including 47 patients (7–35 years), IgE and IgG4 binding to peptide epitopes of caseins and β-lactoglobulin were analyzed before and after immunotherapy. OIT was shown to significantly modify IgE and IgG4 binding to milk allergen epitopes. The study suggests that the analysis of peptide binding epitopes at baseline could be used to predict therapy outcome (121).

Overall, most AIT studies investigating sIgE have shown an increase in sIgE level in the beginning of therapy, followed by a decrease during prolonged therapy. Data also suggest that low sIgE and sIgE/total IgE can predict successful treatment response including SU. Total IgE has consistently shown no significant changes during AIT levels or correlation with clinical response to AIT. Peptide epitopes repertoire and binding seem to be modified during AIT, and this is an area of great interest to understand and predict therapy outcome.

### IgG(4)

In healthy individuals IgG4 is the least abundant IgG isotype in circulation. It has been extensively studied in the context of AIT because of its potential protective effect against allergies. IgG4 is considered an anti-inflammatory antibody isotype because, unlike IgG1, IgG2 and IgG3, it cannot activate the classical complement pathway (122, 123). In addition, IgG4 can undergo a process called Fab arm exchange, during which Fab arms are exchanged between different IgG4 molecules by swapping a heavy chain and attached a light chain (124). This results in the formation of functionally monovalent IgG4 antibodies that are bispecific because they are made up of the heavy chains derived from two different IgG4 antibodies that contain the variable regions. As a result, these antibodies have a severely impaired capacity to form immune complexes and may inhibit the immune complex formation by other isotypes. The production of allergen-specific IgG4 is strongly induced in response to prolonged exposure to high doses of soluble protein antigens (125). Mechanistic studies on the regulation of IgG4 production are hampered by the fact that no functional analog of IgG4 exists in mice.

Changes in concentrations of specific IgG4 have been assessed during OIT with CM (72, 126), peanut (38, 52, 58, 63, 99, 103, 119, 127–131), egg (69, 105, 106, 132–136), and wheat (49, 127). Most of these studies were RCT or pilot studies involving small numbers of patients, with or without placebo arms, and some used omalizumab or probiotics as “adjuvants”. All of these studies except one (103) reported an increase of specific IgG4 concentrations and/or IgG4/IgE ratio during or after OIT. Increases in specific IgG4 concentrations were directly associated to successfully consumed dose (49) and SU (69, 136) and IgG blocking activity was correlated with SU (131). These results tend to relate the IgG4 increase to clinical outcomes of OIT. Moreover, some studies evidenced that higher IgG4/IgE ratio to some allergen components at baseline may be predictive of OIT-induced SU (63, 132).

All but one study demonstrated that allergen-specific IgG antibodies with new antigen specificities developed during AIT (103). Some studies also suggest that specific IgG4 levels are associated with successful up-dosing during AIT and SU. The IgG4/IgE ratio may be more important than the absolute quantity of IgG4 in relation to clinical outcomes (63, 91, 121, 132). No direct correlation between IgG4 concentrations and allergen induced basophil activation has been found (99). This could indicate that other inhibitory factors are involved (such as IgG1, IgA), and also that the inhibitory activity of OIT-induced IgG may be more important than their absolute quantities (137). It should be noted that the effect of IgG blocking activity is dependent on the epitopes that are targeted by these antibodies. IgG that target the same epitopes as IgE are more likely to prevent IgE binding than IgG that target other epitopes, especially if they have a higher affinity for their target than IgE (138). Assessment of the blocking activity using *in vitro* models of facilitated antigen presentation (139) and BAT (91, 99, 131, 140), may greatly improve the outcomes values of this immunological parameter in relation to clinical response to OIT.

### IgA

IgA is an antibody that plays a crucial role in the immune function of mucous membranes (141). IgA has two subclasses (IgA1 and IgA2) and it can be produced as monomeric and dimeric forms. The monomeric molecules are predominantly of the IgA1 subclass, being produced mainly in the bone marrow, while in external secretions most of the IgA, produced locally in mucosal tissues, occurs in the polymeric configuration with a relatively increased proportion of IgA2 molecules (142). The major difference between IgA1 and IgA2 resides in the hinge region that lies between the two Fab arms and the Fc region (143). While IgA1 predominates in serum (~80%), IgA2 percentages are higher in secretions (~35%). The IgA dimeric form is the most prevalent and it is also called secretory IgA (sIgA). sIgA is the main immunoglobulin found in mucous secretions, including tears, saliva, sweat, colostrum, and secretions from the genitourinary tract, gastrointestinal tract, prostate and respiratory epithelium, although it is also found at small amounts in blood.

Our current knowledge suggests a regulatory role of IgA responses in allergy and it has been proposed as a biomarker to assess the effectiveness of food AIT (137). Several studies using peanut (144), CM (116, 145), and egg (132, 146, 147) have assessed allergen-specific IgA before, during and after OIT. One CM OIT study of 40 patients (6–17 years) found that high specific IgA and IgG-subclasses prior to OIT, appear to predict failure to achieve desensitization (145) but this is contradicted by a CM OIT study of 15 patients (6–16 years) showing that high levels of IgA before OIT were observed in responders (116). Several studies showed a significant increase in food-specific IgA and IgA2 during and directly after OIT, which were associated with clinical response to OIT (116, 132, 146) following a decrease in IgA and IgA2 levels during maintenance phase (116). In another study, the levels of specific IgA and sIgA from saliva and serum were investigated in 17 patients (1–11 years) undergoing either peanut SLIT or placebo, at baseline and at the time of the challenge. Salivary and serum levels of peanut-specific IgA increased significantly for subjects receiving SLIT, but not for placebo (144). The same study suggested that salivary IgA could be a prospective biomarker to follow throughout therapy, being potentially useful in defining the efficacy of therapy and in monitoring the progress of the treatment (144).

Most of the available literature suggests that increasing IgA levels might be indicative of a positive response to AIT treatments. However, some contrasting data were reported, in 52 children (5–16 years) with persistent egg allergy who underwent either OIT (N=28) or avoidance (N=24), the serum ovalbumin-sIgA and ovomucoid-sIgA did not change significantly over time, suggesting that none of the serum IgA seems to be associated with induced or natural tolerance to egg allergens (147).

In general, IgA levels of food allergic patients are lower compared to control groups. Overall, most AIT studies investigating IgA have shown an increase during therapy. However, there is a lack of data with respect to the clinical value of IgA assessments for predicting treatment response and SU.

## T Cell Responses

T cells play a key role for the immune response of the host upon ingestion of foods both in the context of obtaining normal homeostasis in a healthy host, i.e. tolerance to food allergens and in the sensitization phase of developing food allergy. Tregs are essential for regulating tolerance to foods as they, upon exposure to food allergens, serve to maintain immune homeostasis *via* secretion of anti-inflammatory mediators such as IL-10 and TGF-β to immune cells and surrounding tissues (148). In line with this, decreased numbers, lower rate of activated Tregs and dysfunctional Tregs with subsequent limited host immune suppressive capability upon ingestion of foods, seem of importance for failure in immune tolerance and susceptibility to develop food allergy (149). Due to the key roles of Tregs and TH2 cells in immune tolerance to foods, it is plausible that the number, phenotype and effector functions of these T cell subsets may serve as biomarkers of FA-AIT treatment response and aid the clinician to predict SU, which would be valuable for the food allergic patient undergoing AIT as it is a long-term treatment with a substantial risk of adverse events and anaphylactic reactions (47).

### Tregs

Despite the expectation that Tregs could be of importance for predicting response to FA-AIT, there has only been few studies addressing this topic. These studies were mostly peanut OIT studies with low numbers of patients and a short follow up in which Treg were analyzed quantitatively and not in terms of suppressive capacity. In a small, DBPC trial investigating the effectiveness of OIT for peanut allergy among 28 patients (1–16 years), the number of CD4+CD25+FOXP3+ Tregs was significantly increased after 1 year of treatment compared to baseline in peanut OIT patients (67). These results of increased Treg numbers were subsequently replicated in a similarly small numbered open-label randomized trial of peanut OIT with 1 year of treatment among 22 patients (7–15 years) (150). Changes in immune suppressive functions of Tregs during and after cessation of food AIT has rarely been studied, but one study investigated this *via* demethylation of FOXP3 CpG sites in Tregs of 23 peanut allergic patients (5–45 years) undergoing OIT compared to 20 age-matched controls on peanut avoidance diet (103). Three months after cessation of peanut OIT the patients developing tolerance, i.e. not reacting to an OFC with peanut, showed hypomethylation of FOXP3 sites compared to patients not tolerant to peanut. However, this increase in immune suppressive functions of Tregs was transient as four of seven initially peanut tolerant patients were no longer peanut tolerant on an OFC and showed increased FOXP3 methylation after an additional 3 months of peanut OIT (103). This return to baseline is in line with findings from an observational study without OIT among 1-year-old peanut allergic (N=14) and peanut sensitized, i.e. tolerant infants (N=15) from the HealthNuts study showing no difference in FOXP3 methylation of Tregs between the groups (151).

One double-blind, placebo-controlled peanut SLIT study of 18 patients (1–11 years) showed no changes in numbers of Tregs or IL-10 production after 1 year of therapy, although a significant proportion of the SLIT-treated children had a positive treatment response and were tolerant to an OFC (110). Treg number or function has not been studied following peanut EPIT treatment, but one double-blind, placebo-controlled study of 74 peanut allergic patients (4–25 years) showed no difference in the percentage of peanut-response CD4+IL10+ T cells following 1 year of treatment (54).

#### TH2 Cells

There are numerous studies investigating secreted levels of type 2 cytokines from TH2 cells during peanut, CM and egg OIT, SLIT, and EPIT, which generally show transient suppressions after therapy. However, only few immunotherapy studies have reported on numbers of TH2 cells, which may be important as a mechanism of successful AIT may include apoptosis of highly differentiated CD27- allergen-specific TH2 cells that was first demonstrated in studies of grass AIT (152). Since then, an allergen-specific T cell subset of memory TH2 cells that are CD4+CD45RO+CD27−CD45RB+CD161+CD49d+ have been described of importance in peanut allergy (153). In a subset of seven of 55 peanut allergic patients aged 4–26 years, who participated in a DBPC peanut OIT it was demonstrated that the number of TH2 cells decreased in patients on OIT therapy compared to placebo (153). Another study of 20 patients (4–18 years), who were enrolled in a peanut OIT showed presence of residual IL-4+ T cells in OIT treated patients initially achieving desensitization based on OFC after 4 months maintenance therapy. This specific T cell population may be re-activated upon peanut exposure and thus serve as a biomarker of the durability of SU following peanut OIT (154).

Overall, most AIT studies investigating Tregs have shown an increase in numbers of Tregs and enhanced, but transient, immunosuppressive Treg functions during therapy. However, there is a lack of data with respect to the clinical value of Treg assessments for predicting treatment response, particularly SU, that is the most important from a patient perspective.

## Responses of Other Cell Types

### B Cells

B cells play a critical role both in the development and persistence of allergies as well as in the induction of allergen tolerance. The contribution of B cells to these processes is primarily related to their unique capacity to produce antibodies. As discussed above, the heavy chain isotype, specificity, and affinity of these antibodies determine their function. B cells are also involved in the regulation of allergic immune responses by means of their interaction with other cell types through production of cytokines and the presentation of allergen-derived peptides to CD4+ T cells.

Allergen-specific B cells can be found in circulation at very low frequencies ranging from <0.03% of class-switched memory B cells in peanut allergic individuals (150, 155) to <0.3% of class-switched memory B cells in bee venom allergic patients (156) and house-dust mite allergic patients (157). The frequencies of circulating Ara h 1- and Ara h 2-specific B cells were found to increase in response to OIT (150, 155). Interestingly, one study reported that the increase of Ara h 2-binding memory B cells peaked after 7 weeks of peanut OIT and then started to decline to levels that were lower than before OIT (150).

Immunosuppressive regulatory B cells can suppress excessive inflammatory response through production of anti-inflammatory cytokines such as IL-10, IL-35, and TGF-β (37, 158). While the role of regulatory B cells has been studied in the context of autoimmune diseases, cancer, transplantation, infections and several allergies (158), no human studies have been performed that focused on the cellular B cell responses in food allergy immunotherapy.

While both qualitative (cell characteristics such as cytokine production and cell surface markers) and quantitative (frequencies of allergen-specific B cells) features of B cells have a potential to be developed as future biomarkers, there are currently no data available that indicate B cell-associated biomarkers that can be used for as biomarkers of food immunotherapy.

### Dendritic Cells

DCs play a key role in initiation of immune responses to food allergens through antigen presentation and driving T cell differentiation. Several mouse studies have been performed to determine the role of DCs in the induction of tolerance to food allergens. Food allergens can be sampled directly from the intestinal lumen by CD103+ DCs (159, 160). After migration to draining lymph nodes, CD103+ DCs can promote development of Tregs through production of TGF-β and retinoic acid (161, 162). These Tregs upregulate the gut homing integrin α4β7 leading to their migration to the lamina propria (163). There are indications that OIT has an effect on circulating DCs, since the peripheral blood DCs after peanut OIT suppressed methylation of Foxp3 in effector T cells (103). Nevertheless, most data on the role of DCs in relation to food allergy is derived from mouse models and immunophenotyping of circulating no distinct DC subsets. So far, this has not led to identification of applicable biomarkers for the use in food OIT.

### Innate Lymphoid Cells

Innate lymphoid cells (ILCs) are subgroups of lymphocytes lacking lineage markers and antigen specific receptors for B and T cells. ILCs have crucial roles in immune defense, regulation of inflammation and tissue remodeling (164). ILCs are primarily tissue resident cells and can be categorized into three groups: group 1 (ILC1 and natural killer (NK) cells, group 2 (ILC2), and group 3 (ILC3 and lymphoid tissue inducer cells), with similar cytokine profiles to TH1, TH2, and Th17, respectively (165). Group-2 ILCs are highly relevant in the context of allergic inflammation, as they share many functional similarities with TH2 cells (165–169). ILC2s, are mainly located in the skin, airway and intestinal mucosa, and can respond to nonspecific stimuli, such as IL-25, IL-33, and thymic stromal lymphopoietin (TSLP) (170).

In food allergy models, IL-33, but not IL-25 or TSLP, was found to be the central cytokine during allergic sensitization (23). IL-33 promotes food anaphylaxis in sensitized mice by targeting mast cells (171) but it also synergizes with intestinal IL-25 production to drive the expansion and activation of ILC2s, which promotes food anaphylaxis (172). Besides, IL-25 stimulates IL-13 production after repeated intragastric allergen challenges (173). It has been postulated that food allergy can be promoted as a result of allergen-specific Treg blockage by stimulated ILC2s (174).

Although ILCs, in particular ILC2s, appear to play a central role in allergic inflammation, so far neither qualitative, nor quantitative measurements of ILCs have resulted in identification of promising FA-AIT-related biomarker targets.

## Cytokines and Other Potential Soluble Biomarkers

Analysis of cytokine production in response to *in vitro* re-stimulation of PBMCs with allergens has also been performed in many studies. OIT studies using peanut, CM, and egg have been reported, in which soluble biomarkers and cytokine measurements were performed in serum or stimulated PMBCs.

Several studies have shown that serum TH2 cytokines are reduced during peanut OIT therapy (67, 112). Moreover, IL-4, IL-5, IL-10, and IL-2 produced by peanut-stimulated PBMCs were reduced in peanut OIT (175). Serum levels of other cytokines that showed significant reductions in response to peanut OIT (49 patients aged 9–23 months) included IL-5, IL-13, and IL-9 (130).

Similarly, a study of 20 egg allergic patients (5–15 years) showed that, at baseline, OVA-re-stimulated PBMCs from allergic patients produced significantly lower levels of IL-10 than healthy controls. After OIT, IL-10 production by allergic patients showed a significant increase (176). While mainly TH2 cytokines are reduced during OIT, one study of 29 patients (1–16 years) with peanut allergy observed that patients treated with OIT had higher levels of IL-10, IL-5, IFN-γ, and TNF-α from peanut-stimulated PBMCs compared with controls (104). Another study of 20 patients (4–18 years) undergoing peanut OIT showed that the changes in cytokine production were based on extended therapy and that pathogenic cytokine producing T cells persisted despite clinical success (154). Transient increases were seen in egg-induced IL-10 and TGF-β levels, and the ratio of TH2/TH1 cytokine production was decreased (177).

In a study of CM allergy in children, β-casein-stimulated PBMCs produced lower IL-5, IL-13, and IL-10 levels at baseline in allergic compared to non-allergic subjects, but after OIT, these differences were not observed (178). In another study, no changes were found in IL-4, IL-5, IL-6, IL-10, and IL-12p70 cytokines or in eosinophil cationic protein, eosinophil-derived neurotoxin, adipokines adiponectin, leptin, and resistin during CM OIT (117). However, serum adipsin was significantly higher in the group who failed OIT, which prompted the suggestion that high serum adipsin after 6 months of increasing OIT doses might predict poor outcome.

A downregulation of the angiogenesis factors platelet-derived growth factor and the vascular endothelial growth factor was found after rapid desensitization and oral immunotherapy in children with CM allergy. Baseline levels of platelet-derived growth factor and vascular endothelial growth factor in the CM allergic patients were already different compared to non-allergic subjects and a significant increase of platelet-derived growth factor and vascular endothelial growth factor was seen in children with a history of anaphylaxis (179). IgE-associated food allergy mechanisms of pathogenesis include cross-linking of mast cell- and basophil-bound IgE and immediate release of inflammatory mediators. Histamine-releasing factor interacts with a subset of IgE molecules and the histamine-releasing factor dimer activates mast cells in a histamine-releasing factor-reactive IgE-dependent manner. Successful OIT, assessed by attaining a level of desensitization without showing unbearable adverse effects, such as refractory gastroenteritis and/or severe anaphylaxis in egg-allergy patients reduced histamine-releasing factor-reactive IgE levels (180).

Besides using biomarkers from sera and *ex vivo* stimulation, there are some metabolites found in the urine, which have potential as soluble biomarkers for FA-AIT. One example is tetranor-PGDM, which is a metabolite of prostaglandin D2 secreted by mast cells. One study showed that urinary tetranor-PGDM concentration at 4 h after food intake is a predictor of the effectiveness of OIT (181).

As it remains unclear whether these markers are predictive of efficacy, computational approaches have been attempted to compare mechanistic features of food allergies during OIT to determine the biological relevance of biomarkers and have led to promising clusters of biomarkers (182).

The lack of reproducibility and/or predictability of these measurements make them difficult to rely upon. A major problem of the use of serum and recall PBMC-stimulated cytokines and other metabolites as predictive biomarkers is the difficulty in selecting the best timing for measuring OIT outcome. These values may have diurnal fluctuation and may be modulated by a myriad of other factors in the subject or their environment. It should also be noted that there are many differences in the execution of the PBMC re-stimulation. Stimulation periods vary from 48 h up to 7 days, some use autologous serum/plasma in the medium and some report LPS-free allergen extracts. These variables can all have impact on the levels of produced cytokines. More research is needed to evaluate these approaches for OIT outcome measures.

## AIT in Combination With Omalizumab

To reduce adverse reactions during AIT, it has been suggested to combine CM OIT with omalizumab treatment (183). In a randomized DBPC study including 57 participants (7–32 years), adverse reactions were found to be reduced during the escalation phase, whereas sIgE to CM increased at month 4 (57). In this study, omalizumab treatment significantly improved the safety of the OIT, but not its efficacy. There was, however, the benefit of a reduced risk of severe adverse effects during OIT. In another CM OIT study including 57 patients (7–35 years), the ability of baseline markers to predict which individuals would benefit from an omalizumab therapy was explored (94). Participants whose basophils reacted at greater extent than a threshold of 40% CM-induced CD63 expression were less likely to have symptoms if they received adjunctive therapy. Thus, omalizumab treatment could be beneficial for patients at high risk of adverse reactions.

Another small study included 14 children (3–13 years) refractory to egg and CM OIT (184). These patients received omalizumab treatment during the escalation phase, it was stopped 2 months after reaching the final dose. Administration of omalizumab permitted a safe and effective desensitization treatment in these patients, however 60% of the CM treated patients and 33% of the egg allergic patients relapsed after omalizumab suspension. Clinical reactivity threshold decreased at 2–4 months after suspending omalizumab.

Although omalizumab is currently only approved for treatment of severe allergic diseases, small pilot studies have shown promising results in conjunction with immunotherapy. Larger studies are needed in order to determine biomarkers that would identify those patients that would benefit the most from combined therapy by substantially reducing adverse effects.

## Patient Related Outcomes

It is important that the patient’s perspective is at the center of new developments in AIT. Identification of predictors (both clinical and psychosocial) could optimize treatment by helping to implement appropriate, suitable and harmonized approaches that will benefit patients and can inform standardization of treatment protocols.

With AIT, the possibility of success must be weighed against risk, for example the annual rate of severe reactions in peanut allergy is 1.6% (185). However, in the clinical setting severe reactions are anticipated, recognized and treated promptly. The context of “expected” reactions in AIT differs from the uncertain but potential accidental reactions with avoidance (186) and caregivers have reported that trial participation decreased their anxiety secondary to experiencing a severe allergic reaction that was promptly treated.

AIT also mitigates severe reactions with accidental exposure and is a major motivator for entering a child into a clinical trial. Thus, when a patient threshold is raised, even if SU is not achieved following treatment, benefit may still accrue. This positive impact may have been reinforced by specialist consultation, personalized information, and interaction with other children with food allergy during treatment.

Patient reported outcome measures, particularly health related quality of life (HRQL) questionnaires, are increasingly being used in AIT trials, as secondary outcome measures (187). HRQL is useful in evaluating new policies, technologies, regulations or clinical practices and for guiding decision making when there is a trade-off between treatment and HRQL (188–190). The measures have ensured that the impact of medical conditions and/or treatments are now being evaluated from the perspective of their impact on the specific patient’s everyday life.

Treatment success in AIT trials, may be defined not only by desensitization or SU but by improved HRQL, or an interaction between the two. Both may depend on tailoring interventions to the characteristics and needs of patients. More data is needed to determine whether the outcomes of SU and desensitization have a different impact on HRQL.

## Discussion

The current literature comprises numerous reports of FA-AIT trials in both pediatric and adult patient populations. The increase in research in the field is dictated by the need to effectively transition from traditional physician guidance on allergen avoidance to active treatment. The ultimate goal of this transition is to improve quality of life and to reduce the severity of allergic reactions in FA patients. In the context of FA-AIT trials, it is important to distinguish between the two major objectives of immunological outcome measurements: 1) To identify outcome measures that have predictive value for the probability of successful AIT that can be assessed at baseline. This may aid in the selection of patients that are more likely to benefit from FA-AIT. 2) To monitor the immune response to the intervention throughout FA-AIT, both in the short-term (i.e. immediately after completion of AIT), and in the long long-term (i.e. assessment of SU). Achieving these objectives remains a challenge, in particular due to the absence of insufficient standardization of outcome measures employed in different clinical trials, and the high degree of uncertainty concerning the informative value of these different outcome measures.

### Immunological Changes During FA-AIT

Reduced basophil and mast cell reactivity are among the earliest changes that occur during AIT. This response typically occurs within the first months of AIT and precedes decreases in allergen specific-IgE levels and therefore cannot be attributed to that. During the initiation phase of FA-AIT, subthreshold dosing induces IgE internalization and actin rearrangement causing hyporesponsive effector cells. Other mechanisms that may drive these early effects on include histamine receptor 2 upregulation in basophils leading to suppression of basophil activation (35), and piecemeal basophil and mast cell degranulation occurring early during AIT, which may decrease their granule content and affect their threshold for activation (191). Most studies reported reduced SPT reactivity after the start of therapy.

Changes in humoral responses during AIT include early increases in allergen-sIgE levels, which are observed during the first weeks and months of AIT, possibly resulting from an initial boost of allergen-specific B cell activation. This is followed by a reduction in allergen-sIgE and an increase in allergen-sIgG4 and IgA during the following months.

T cell responses have traditionally been studied in FA-AIT with an emphasis on cytokine production by *in vitro* allergen-restimulated PBMCs. No consistent results emerged from these experiments, and changes in the production of TH2, TH1, and Treg-related cytokines differed substantially between studies. Increases in Treg frequencies were observed 1 year after the start of peanut OIT (67, 150), while a temporary increase in the suppressive function of Tregs was reported in a small group of patients, suggesting a possible effect of FA-AIT on Treg suppressive capacity (103). On the other hand, it appears that that allergen-specific TH2a cells decrease during peanut OIT (153).

Changes in frequency and/or functionality of other cell types such as B cell, DC, and ILC subsets have not been studied in detail in the context of FA-AIT and their potential roles remain to be determined.

It should be emphasized that FA-AIT-induced immunologic changes may be temporary, even while patients receive continued maintenance treatment, highlighting inter-individual variability in immune suppression and clinical response.

The development of new technologies such as single-cell-omics approaches and high-dimensional platforms for detection of cell surface markers, allergen-specific antibodies as well as soluble biomarkers combined with sophisticated bioinformatics approaches, will enable the exploration of these mechanisms at an unprecedented level of detail potentially revealing novel candidates that may be used as immunological outcome measures in FA-AIT.

### The Value of Measuring Immunological Outcomes in FA-AIT

In this review, we critically appraised the utility of different immunological parameters as outcome measures in FA-AIT clinical trials. **Supplementary Table 1** provides an overview of the FA-AIT trials discussed. Of the 38 FA-AIT studies listed in **Supplementary Table 1**, 16 studies calculated a correlation between one/more biomarkers and a clinical outcome (e.g., response vs. no response to AIT) (53, 63, 94, 98–102, 115–117, 132, 145–147, 153). Based on these studies, we have summarized the following recommendations for biomarkers to be measured before, during and after FA-AIT. The biomarkers are also listed in **Table 1**.

**Table 1:** Immunological markers and their potential predictive value in food AIT.

Biomarker	Predictor for successful AIT*	Marker during/at the end of AIT	Marker for SU
**Functional tests**			
SPT	+ (↓)	+++ (↓)	+(↓)
CD63 (BAT)	+ (↓)	+++ (↓)	+ (↓)
**Humoral responses**			
sIgE	+ (↓)	+++ (↑→↓) Early increase followed by decrease	+ (↓)
sIgE/total IgE ratio	+ (↓)	(+)(↓)	++ (↓)
Specific IgG4	+ (↑)	+++ (↑)	++ (↑)
IgG4/IgE ratio	(+)(↑)	+++ (↑)	++ (↑)
IgA	+/- (↑ and ↓)	+++ (↑)	(+) (↑)

*The smaller (↓) or higher (↑) the better chance of success.

+indicates the biomarker changes during AIT or differs between responders vs. no-responders.

-indicates the biomarker does not change during AIT or does not differ between responders vs. no-responders

+, one study/weak evidence.

++, two studies/medium level of evidence.

+++, at least three studies showing the same thing/strong evidence.

This table only lists immunological markers that were reported to have predictive value for desensitization (successful AIT), sustained unresponsiveness (SU), or that showed significant changers during AIT in at least two studies.

The immunological parameters were grouped into four main categories: 1) functional tests, 2) humoral responses, 3) cellular responses, 4) cytokines, and other potential soluble biomarkers.

### Functional Immunological Tests

BAT shows promise as a predictive biomarker for successful FA-AIT. In most studies, BAT was assessed by measuring the frequency of CD63+ basophils after *in vitro* allergen exposure. Several studies found that lower allergen-induced CD63 expression at baseline correlated with successful AIT (63, 99) and also with obtaining SU (98, 99). Most studies reported that allergen-induced CD63 expression was generally reduced during FA-AIT, both in patients showing clinical response to FA-AIT and non-responders (6, 53, 98, 99, 192) except in one study where no change was observed (100).

The magnitude of the SPT response at baseline was not reported in any of the studies as having predictive value with respect to the clinical outcome of FA-AIT. SPT may have value for monitoring the immune response during therapy, as a SPT response at the end of the AIT was associated with successful AIT in several studies (101, 102), however one study found no association between SPT and outcome of OIT (100).

### Humoral Responses

FA-AIT studies in which allergen-specific IgE levels were determined have yielded varying results. While some studies found no correlation (54, 147), most studies observed a negative correlation between specific IgE levels and success of OIT (99, 100, 132, 145, 147). Total IgE consistently showed no correlation with success of OIT (53, 116) while a lower specific IgE:total IgE-ratio at baseline correlated with SU (94, 99).

Most studies reported that increased specific IgG4 levels were associated with a successful outcome (53, 132, 145), but one study observed no difference between groups (146) and another study even found a reverse association (99). However, an increased ratio of sIgG4:sIgE was consistently associated with successful outcome in several studies (99, 116, 132, 146).

Studies in which allergen-specific IgA levels were determined found contradictory results for baseline levels of specific IgA and their correlation with a successful outcome of AIT (116, 145), However, it was consistently found that allergen-specific IgA levels increased during AIT (116) and this increase was often more pronounced in responders than in non-responders (132, 145, 146).

### Cellular Responses

There is a lack of data with respect to the clinical value of Treg assessments for predicting response to OIT, particularly SU. It is a major limitation that all evidence is based on small studies with short follow-up periods, which are predominantly peanut OIT trials. Therefore, although Treg assessment seems promising to determine response to FA-AIT, there is a need for large-scale, long-term studies to properly assess the value of Tregs. Most promising is the assessment of TH2a cells and residual IL-4+ T cells that may be clinically useful for monitoring response to peanut OIT and durability of SU after peanut OIT, respectively. Further studies of TH2 cells are needed to replicate these findings and confirm a clinical applicability.

The role of other cell types such as B cell, DC, and ILC subsets have not been studied in the context of predicting outcome of FA-AIT.

### Cytokines and Other Potential Soluble Biomarkers

The lack of reproducibility and/or predictability of these measurements make them difficult to rely upon. Only one study looked at cytokines in relation to clinical outcome and found no correlation with development of tolerance (115). As it remains unclear whether these markers are predictive of efficacy, machine learning approaches have been attempted to compare mechanistic features of food allergies during OIT to determine the biological relevance of biomarkers and have led to promising clusters of biomarkers (182).

On the basis of currently available literature we conclude that the immunological parameters with the strongest evidence pertaining to both 1) predicting probability of successful FA-AIT and 2) monitoring the immune response to the intervention, both in the short-term (i.e. immediately after completion of FA-AIT), and in the long long-term (i.e. assessment of SU), are functional tests: SPT and BAT, and humoral responses: sIgE, sIgE:total IgE-ratio, sIgG4, sIgG4:IgE ratio, and IgA.

## The COMFA Consortium Members

Karine Adel-Patient, Flore Amat, Corina Bocsan, Erna Botjes, Robert Boyle, Dominique Bullens, Bo Chawes, Costas Christophi, Tanja Cirkovic-Velikcovic, Joana Costa, Audrey DunnGalvin, Gillian DunnGalvin, Michelle Epstein, Johan Garssen, Jon Genuneit, Christiane Hilger, Despo Ierodiakonou, Thuy-My Le, Ines Marino, Vildan Mevsim, Dragan Mijakoski, Daniel Munblit, Nikita Nekliudov, Cevdet Özdemir, Pedro Rodrigues, Patrick Sammut, Ann-Marie Malby Schoos, Martin Sørensen, Sasho Stoleski, Eva Stylianou, Paul Turner, Willem van de Veen, Michelle Helena Van Velthoven, Dominique Vandekerchove, Kitty Verhoeckx, Mihaela Zidarn, Anna Andrałojć, Christian Apfelbacher, Gastone Castellani, Joanne Chalmers, Ilya Korsunsky, Annette Kuehn, Sarah Ninane, Diego Peroni, Pablo Rodriguez Del Rio, Jelena Roomet, Hania Szajewska, Zsolt Szepfalusi, Martin Teufel, Mirjana Turkalj, Christina West.

## Author Contributions

All authors contributed to drafting this review and approved its final version.

## Funding

COMFA is funded by the European Cooperation in Science and Technology (COST) (CA18227). The COST Association had no role in the design and management of the COMFA projects. DB received funding from Fund for Scientific Research (FWO)Flanders, TBM-project T001419N; DB is recipient of Senior Clinical Investigatorship from Fund for Scientific Research (FWO) Flanders. JC received funding from FCT (Fundação para a Ciência e Tecnologia) under the Partnership Agreement UIDB 50006/2020 and project AlleRiskAssess—PTDC/BAAAGR/31720/2017. CH and AK received funding from the Luxembourg National Research Fund on PRIDE program grants PRIDE/11012546/NEXTIMMUNE and PRIDE17/11823097/MICROH. DMunblit received funding from«5–100» Russian Academic Excellence Project and was involved in expert activities within ILSI Europe expert group.

## Conflict of Interest

ADG received funding and consulting fees from Aimmune and DBV. KK was employed by Danone Nutricia research. JG is part time employee of Nutricia research. DMunblit has given paid lectures for Merck Sharp & Dohme and Bayer.

The remaining authors declare that the research was conducted in the absence of any commercial or financial relationships that could be construed as a potential conflict of interest.

## Abbreviations

AIT, allergen-specific immunotherapy; BAT, basophil activation test; CM, cow’s milk; DBPCFC, double blind placebo controlled food challenge; EPIT, epicutaneous immunotherapy; FA, food allergy; FA-AIT, food allergen immunotherapy; fMLP, N-Formyl-methionyl-leucyl-phenylalanine; HRQL, health related quality of life; Ig, immunoglobulin; MAT, mast cell activation test; OFC, oral food challenge; OIT, oral immunotherapy; RCT, randomized clinical trials; SCIT, subcutaneous immunotherapy; sIgE, specific IgE; SLIT, sublingual immunotherapy; SPT, skin prick test; SU, sustained unresponsiveness; Treg, regulatory T cell.
